# Predictive Value of Preoperative Maximum Standardized Uptake Value (SUVmax) in Patients with Advanced Gastric Cancer

**DOI:** 10.3390/biomedicines13030554

**Published:** 2025-02-21

**Authors:** Yinwen Sun, Xiangfei Sun, Ran Xiong, Chao Li, Yuning Zhou, Wenchao Jiang, Hongshan Wang, Xiaodong Gao

**Affiliations:** 1 Department of General Surgery, Zhongshan Hospital, Fudan University School of Medicine, Shanghai 200032, China; 23211210061@m.fudan.edu.cn (Y.S.); sun.xiangfei@zs-hospital.sh.cn (X.S.); 24211210056@m.fudan.edu.cn (R.X.); 23111210155@m.fudan.edu.cn (C.L.); zhouyuning418@163.com (Y.Z.); 22211210040@m.fudan.edu.cn (W.J.); 2 Department of General Surgery, Zhongshan Hospital Wusong Branch, Fudan University, Shanghai 200940, China; 3 Baoshan Cancer Center, Baoshan District, Shanghai 200940, China

**Keywords:** maximum standardized uptake value, GLUT-1, advanced gastric cancer, prognosis

## Abstract

**Background:** This study aimed to investigate the clinical and prognostic significance of preoperative maximum standardized uptake value (SUVmax) and GLUT-1 expression in patients with advanced gastric cancer (AGC). **Methods:** Medical records of patients who were diagnosed with AGC between 2018 and 2020 at Zhongshan Hospital of Fudan University (Shanghai, China) were retrospectively analyzed. Finally, 182 patients were enrolled, and for each patient, SUVmax was calculated for the primary lesion on PET/CT prior to curative surgery. A total of 165 clinical tissue specimens were collected for immunohistochemical analysis of GLUT-1 expression. **Results:** A total of 182 patients were divided into two groups based on their SUVmax values. The low SUVmax group comprised 92 patients. Patients with low SUVmax tended to be younger and included a higher proportion of women, with their primary tumors typically smaller or in earlier TNM stages. The median follow-up time was 52 months. The 1-, 3-, and 5-year progression-free survival (PFS) rates were 90.7%, 71.4%, and 67.0%, respectively. Among them, 33 patients experienced recurrence and metastasis, and 40 ultimately died. Log-rank analysis revealed that the low SUVmax group exhibited superior progression-free survival (PFS) and overall survival (OS). Multivariate analysis indicated that, for AGC without preoperative treatment, later stage (stage III) was independently correlated with a higher risk of recurrence (HR = 3.049; 95%CI = 1.076–8.639; *p* = 0.036), while the low SUVmax group exhibited a reduced risk of recurrence and mortality compared with the high SUVmax group (HR = 0.565; 95%CI = 0.326–0.979; *p* = 0.042). **Conclusions:** The clinicopathological characteristics of patients with AGC with different SUVmax values appeared significantly different. Tumor stage and SUVmax were found as independent factors affecting postoperative recurrence and death of patients with AGC.

## 1. Introduction

Gastric cancer is one of the most common gastrointestinal malignancies, in which more than 968,000 new cases and nearly 660,000 deaths were reported in 2022, ranking fifth in terms of both incidence and mortality rates worldwide [1]. Surgery is the core treatment for gastric cancer, and patients with early gastric cancer benefit greatly from complete resection surgery. However, patients with stage III tumors undergoing surgery have a dismal 5-year survival rate of 18–50% [2].

^18^F-fluorodeoxyglucose positron emission tomography–computed tomography (^18^F-FDG PET/CT) has demonstrated its potential for the diagnosis and preoperative staging of gastric cancer [3], which promotes restaging recurrent gastric cancer to predict prognosis [4]. The uptake of FDG in the tumor is directly proportional to the metabolic rate of living tumor cells, thereby potentially reflecting the biological aggressiveness of the tumor. The FDG uptake can be quantified as the standardized uptake value (SUV), which is the tissue concentration of FDG in the region of interest divided by the injected dose normalized by body weight. The maximum SUV (SUVmax) has been widely accepted as one of the simplest and most accurate parameters to apply in clinical practice [5], which can intuitively indicate the glucose metabolism characteristics of tumors. Previous studies have demonstrated that SUVmax is associated with biological features, such as histological grade [6], glucose transporter expression [7], tumor hypoxia, and angiogenesis [8,9] in various tumors, contributing to tumor growth, cancer recurrence, treatment resistance, and metastasis. SUVmax has been proposed as a predictor of tumor prognosis. The higher the SUVmax of gastric lymphoma and colorectal cancer imaged by ^18^F-FDG PET/CT before treatment [10,11], the worse the prognosis. ^18^F-FDG uptake holds significant prognostic value in neuroendocrine neoplasms of gastroenteropancreatic origin [12], and SUVmax even surpasses pTNM stage or other indicators in predicting prognosis [13]. SUVmax is also associated with the prognosis of patients with gastric cancer, while due to individual differences in patients and limited follow-up time, the applicability of SUVmax in survival analysis of gastric cancer remains to be investigated [14].

GLUT-1 was chosen as the primary focus due to its pivotal role in glucose metabolism and FDG uptake. It has been extensively validated as a key biomarker for FDG avidity in tumors such as pancreatic [15] and colorectal [16] cancers. By adopting GLUT-1, we aim to systematically evaluate the correlation between SUVmax (a clinically accessible parameter from PET/CT) and tumor metabolic markers, thereby strengthening the biological relevance of imaging-derived metrics.

## 2. Materials and Methods

### 2.1. Patients’ Selection

Patients with AGC treated consecutively at Zhongshan Hospital of Fudan University (Shanghai, China) between January 2018 and December 2020 were retrospectively enrolled. The inclusion criteria were summarized as follows: (1) histologically proven AGC confirmed by postoperative pathological specimens; (2) the history of undergoing gastrectomy shortly after diagnosis; (3) the history of undergoing preoperative ^18^F-FDG PET/CT examination; and (4) the availability of complete medical history and clinicopathological data. The exclusion criteria were summarized as follows: (1) the history of undergoing preoperative treatments, such as chemotherapy; (2) the tumor invaded other organs or was combined with other tumors; and (3) the presence of neuroendocrine tumors. Data from 182 patients constituted the final clinical database. Informed consent was obtained from all patients, and the study was undertaken in compliance with the requirements of the Declaration of Helsinki. This study was approved by the Institutional Review Board (IRB) of Zhongshan Hospital Fudan University. Written informed consent had been obtained from all participants.

**Figure 1:**
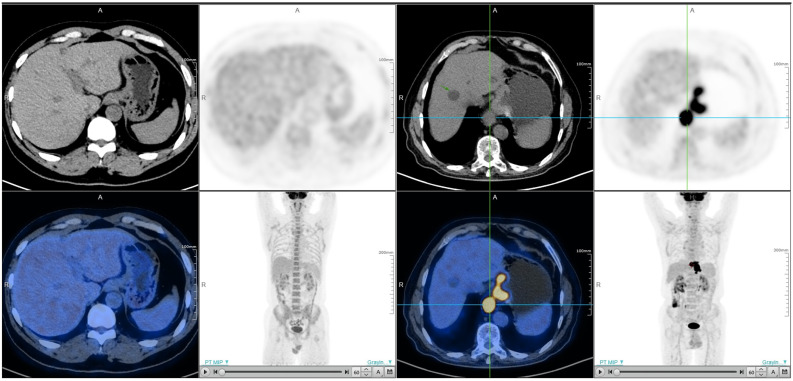
The comparison of ^18^F-FDG PET/CT images between a patient in the low SUVmax group and a patient in the high SUVmax group (the left one with an SUVmax of 1.5, and the right one with an SUVmax of 52.9).

There are differences in the definition of SUVmax value across different studies. While an SUVmax threshold of 2.5 has been proposed to distinguish between benign and malignant lesions [17], this approach overlooks clinically relevant information about tumor biology that can be gleaned from an FDG PET examination. Salaun et al. [18] demonstrated that an SUVmax of less than 4 is not mainly associated with gastroesophageal neoplasia. Kim et al. [19] concluded that all primary tumors demonstrating detectable ^18^F-FDG uptake in the study of AGC exhibited an SUVmax exceeding 3.0 [20]. They defined an SUVmax below 4 as indicative of low tumor ^18^F-FDG avidity. Based on the data we collected, 4 was the median SUVmax of 182 patients, and we used this as the threshold to divide the patients into two groups. A total of 92 patients with AGC with an SUVmax of no more than 4 were categorized into the low SUVmax group, signifying low tumor ^18^F-FDG avidity and diminished glucose metabolism. Conversely, 90 patients with an SUVmax > 4 were allocated to the high SUVmax group. Notably, ^18^F-FDG PET/CT images of patients in the low SUVmax group scarcely revealed tumor presence versus those of patients in the high SUVmax group (Figure 1).

### 2.2. Immunohistochemical Examinations

We finally collected the paraffin-embedded cancer tissue blocks of 165 patients. Representative regions of gastric cancer were marked on specific paraffin blocks according to the results of HE staining. Altogether 165 paraffin-embedded cancer tissue blocks were collected, and samples (2 mm × 6 mm) were acquired by inserting tissue array needles and then aligned on blank paraffin blocks to make a tissue microarray (TMA). IHC was performed on 5-micron slice TMA on a fully automated immunohistochemistry machine (Leica Bond-Max, Leica Biosystems, Shanghai, China), with the antibody of GLUT-1 (1:1000; Servicebio; Wuhan, China; Cat number: GB113495). A fully automatic digital slice scanning system (Leica Aperio AT2, Leica Biosystems, Shanghai, China) was used to scan the IHC staining images of each TMA. According to the intensity of the staining, the positive reaction of GLUT-1 was scored into four grades: 0 (negative), 1 (low), 2 (moderate), and 3 (high). The percentage of GLUT-1-positive cells was also scored into five grades: 0 (0%), 1 (<10%), 2 (10–50%), 3 (51–80%), and 4 (>80%). The immunoreactive score (IRS) gives a range of 0–12 as a product of multiplication between the intensity and percentage scores. To classify GLUT-1 expression, we employed a median-based cutoff approach. The median IRS value across the cohort was determined, and a cutoff of IRS > 3 was established to define the GLUT-1 high expression group, while patients with IRS ≤ 3 were classified as the GLUT-1 low expression group.

### 2.3. Observational Indicators

The data of age, gender, primary and metastatic sites, tumor size, pathological reports, immunohistochemical results, and postoperative adjuvant therapy were collected. SUVmax values were extracted from the reports of ^18^F-FDG PET/CT scan. Data on recurrence and survival were collected from 182 patients and analyzed to identify prognostic risk factors.

### 2.4. Follow-Up Examination

The endpoints were progression-free survival (PFS) and overall survival (OS), which were obtained through regular outpatient follow-up and telephone follow-up. PFS was defined as the duration from the primary tumor resection to the initial confirmation of tumor progression or the last follow-up. OS was defined as the time from primary tumor resection to death or the last follow-up. The last follow-up time was March 2024.

### 2.5. Statistical Analysis

All measurement data were statistically described as mean ± standard deviation ($\bar{x}$ ± SD) or median (quartiles) [M (Qr)] according to their normal distribution. Group comparisons were made using Student’s t-test or the Mann–Whitney U test. Count data were statistically described as number of cases (%), and differences between groups were analyzed using the Chi-square test or Fisher’s exact test. Univariate and multivariate logistic regression analyses of clinicopathological factors were conducted to assess the association of PFS or OS with SUVmax. Kaplan–Meier method with log-rank test and Cox proportional hazards model were utilized for survival analysis. PFS and OS curves were plotted using Kaplan–Meier estimates, and the difference between survival curves was tested using log-rank test. Two-sided *p* < 0.05 was considered statistically significant. Statistical analysis was performed using SPSS 26.0 (IBM Corp., Armonk, NY, USA) and R 4.3.0 (http://www.r-project.org/) (accessed on 10 May 2024) software.

## 3. Results

### 3.1. GLUT-1 Protein Expression in Patients with Different Levels of SUVmax

**Figure 2:**
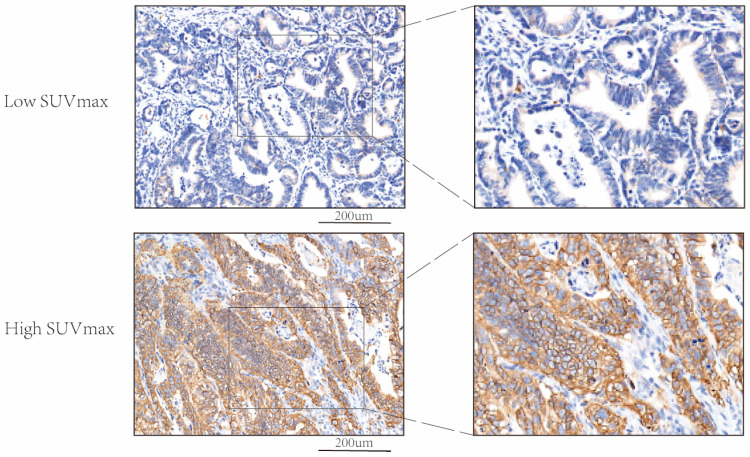
The comparison of two representative IHC images of GLUT-1 expression between a patient in the low SUVmax group and a patient in the high SUVmax group.

**Figure 3:**
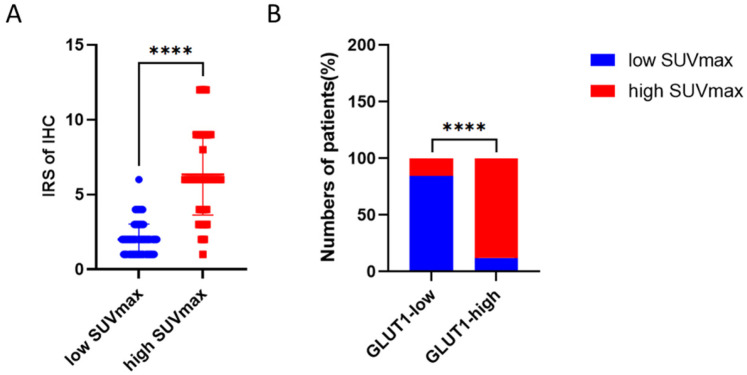
The immunoreactive score (IRS) indicated that patients in high SUVmax group tend to have a high level of GLUT-1 expression (**A**), and the GLUT-1-high group contained a significantly higher proportion of patients with high SUVmax (**B**). ****: *p* < 0.0001.

Immunohistochemistry analysis was performed to examine the protein expression of GLUT-1 in patients with different SUVmax. As shown in Figure 2, GLUT-1 was mainly found in the cell membrane, and its protein expression is elevated in patients with high SUVmax. Figure 3 showed that patients with higher SUVmax tend to have an elevated expression of GLUT-1.

### 3.2. Baseline Clinicopathological Data

A total of 182 patients (107 men and 75 women) were included in this study. Patients’ median age was 64 (range: 25–90) years old. Table 1 presents a summary of patients’ pretreatment characteristics. There were significant differences in clinicopathological characteristics between the two groups. Patients categorized in the low SUVmax group tended to be younger and comprised a larger proportion of women. Their primary tumors were generally smaller or at earlier TNM stages. Additionally, they exhibited a higher prevalence of diffuse type, signet-ring cell carcinoma (SRCC) component, or HER2 negativity. Conversely, the MSI-H type was predominantly concentrated in the high SUVmax group.

### 3.3. Prognostic Analysis

The survival data of the 182 patients were analyzed. After radical gastrectomy, 92 patients underwent postoperative adjuvant chemotherapy regimens, such as S-1 and oxaliplatin (SOX), XELOX (capecitabine (Xeloda) plus oxaliplatin), etc., including 43 patients in the high SUVmax group and 49 patients in the low SUVmax group. At the deadline, 121 (66.5%) patients remained alive and were followed up for a median of 52 (5–74) months. The 1-, 3-, and 5-year PFS rates were 90.7%, 71.4%, and 67.0%, respectively. During the follow-up period, 61 patients developed disease progression. Furthermore, 17 patients had distant metastasis in the liver, bones, ovaries, lungs, and colon, and 8 patients developed metastases in the abdomen and pelvis, and eventually, 41 of them succumbed to the disease. Among 61 patients with tumor progression, 36 patients received postoperative adjuvant chemotherapy. In the low SUVmax group, among patients who received postoperative adjuvant chemotherapy, 16 out of 49 patients experienced disease progression, resulting in 9 deaths. Conversely, in the high SUVmax group, 20 out of 43 patients on postoperative adjuvant chemotherapy had disease progression, leading to 13 deaths.

**Figure 4:**
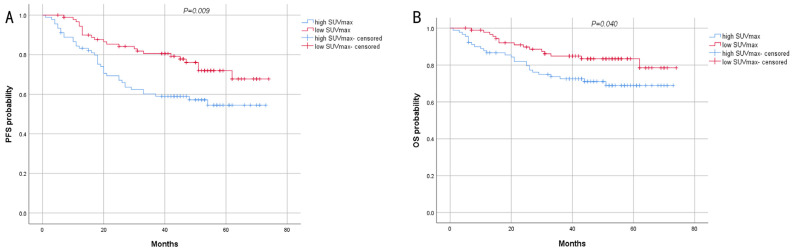
Kaplan–Meier survival analysis of PFS (**A**) and OS (**B**) for patients with different SUVmax.

**Figure 5:**
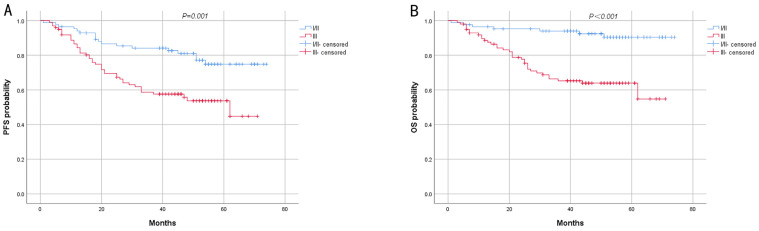
Kaplan–Meier survival analysis of PFS (**A**) and OS (**B**) for patients with different TNM stages.

**Figure 6:**
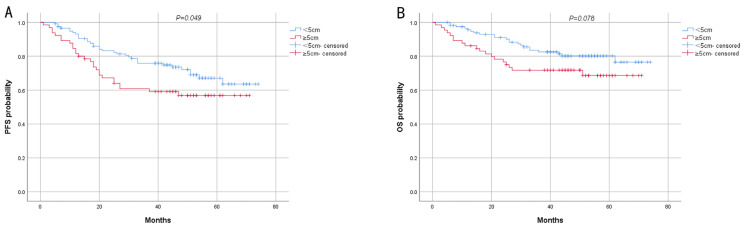
Kaplan–Meier survival analysis of PFS (**A**) and OS (**B**) for patients with different tumor sizes.

The results of log-rank analysis and survival curves showed that early stage (stage I and II) and low SUVmax were associated with longer PFS and OS (Figure 4 and Figure 5), and the difference was statistically significant. Tumor size <5 cm only exhibited a significant difference in PFS between the two groups (Figure 6). Other pathological characteristics had no significant effect on PFS or OS. Subgroup analysis of the two groups indicated that SUVmax had a consistent effect on prognosis across most subgroups. However, for patients with advanced-stage disease, large tumor size, or positive HER2 status, SUVmax minimally influenced prognosis (Table 2). Univariate analysis of PFS revealed that SUVmax, TNM stage, lymph node metastasis, type of resection, and adjuvant chemotherapy were significant factors influencing PFS. Multivariate logistic regression analysis indicated that late TNM stage and high SUVmax were independent risk factors for the progression of AGC (Table 3). Prognostic analysis revealed that the prognosis of high SUVmax group was worse than that of low SUVmax group.

## 4. Discussion

“Warburg effect”, also named “aerobic glycolysis”, can result in the increased glucose consumption, enhanced glycolytic activity, and lactate accumulation, which are the key characteristics of tumor cells [21]. PET/CT relies on the increased glucose uptake of tumors, with ^18^F-FDG serving as a tracer to reflect glucose metabolism. Consequently, ^18^F-FDG PET/CT is widely utilized in tumor diagnosis and monitoring, including gastric cancer. Compared with contrast-enhanced CT (CECT) and endoscopic ultrasound, PET/CT has higher specificity, while it lacks sensitivity and accuracy advantages [22]. This discrepancy might result from the low glucose metabolism of some tumors, in which PET/CT fails to detect, or from indistinguishable SUVmax values. The role of SUVmax in the prognosis of gastric cancer remains controversial [14], and different selection criteria may lead to different conclusions. To explore the predictive value of SUVmax in resectable gastric cancer prognosis and to make postoperative treatment decisions, a comparison was made between the clinical characteristics and prognosis of 92 patients with low SUVmax and 90 patients with high SUVmax. Notably, the present study concentrated on patients with AGC who had not undergone neoadjuvant therapy prior to surgery, as early gastric cancer mainly exhibits low FDG uptake owing to smaller lesions and markedly better prognosis [23]. We excluded patients who underwent neoadjuvant therapy because neoadjuvant therapy may substantially alter tumor biology and FDG uptake patterns, which would introduce confounding variables and make it challenging to isolate the true predictive value of SUVmax in untreated AGC. However, investigating the impact of neoadjuvant therapy on SUVmax and its prognostic value is an important area for future research.

PET/CT imaging of gastric cancer may be affected by several factors, and the ability of FDG uptake may be associated with the depth of tumor invasion and histological subtype [24]. The present study indicated that there were significant differences in T stage and Lauren classification distribution between the two groups. Alakus et al. demonstrated that the SUVmax of diffuse-type gastric cancer and SRCC was significantly lower [25], and the present study also found that their proportion was larger in the low SUVmax group. Alakus et al. suggested that the low expression level of glucose transporter 1 (GLUT-1) may be the cause of the low FDG uptake in SRCC. [25] Shi et al. reported that SLC2A1 (the gene encoding GLUT-1) expression level was significantly upregulated in patients with gastric cancer [26], which could be significantly correlated with invasion depth and clinical stage. Its high expression level can promote tumor growth in vivo, enhance glucose utilization, and also contribute to tumor metastasis. GLUT-1 expression level was also elevated in benign gastric schwannoma with high FDG uptake [27] and primary gastric lymphoma [28]. However, Takebayashi et al. demonstrated that SUV was not correlated with GLUT-1 expression level [23], while it was correlated with hypoxia-inducible factor-1 HIF-1α expression level, and FDG accumulation may represent tissue hypoxia rather than glucose transport activity. In our study, the expression levels of glucose transporters were measured in the two groups, and GLUT-1 expression is likely to be a significant reason for the difference in FDG uptake.

In Lee et al.’s analysis of histopathological subtypes [29], although ^18^F-FDG uptake in gastric cancer emerged as an independent and notable prognostic factor for tumor recurrence, its significance was only marginal in patients with signet-ring cell carcinoma or mucinous carcinoma. However, findings of the present study align with those of Pak et al., suggesting that a higher SUVmax indicates a more aggressive tumor biology in advanced SRCC and is correlated with a poorer prognosis [30].

The relationship between HER2 status and SUVmax remains controversial. Romulo Celli et al. found that there was no statistically significant difference in SUVmax between HER2-positive and HER2-negative patients [31]. However, Park et al. demonstrated that, for patients with inoperable AGC, HER2-positive gastric cancers exhibited higher SUVmax compared with HER2-negative ones [32]. The present study further confirmed a higher proportion of HER2-positive patients in the high SUVmax group among patients with operable AGC. The subgroup analysis revealed that HER2-positive patients in the low SUVmax group may have a worse prognosis than HER2-positive patients in the high SUVmax group, while the conclusions may need to be verified in studies with larger samples. Similarly, subgroup analysis indicated that, for patients with late-stage or large tumors, a low SUVmax did not necessarily represent better prognosis. This discrepancy may result from the fact that HER2 positivity, late-stage disease, and large tumor size are indicative of high malignancy in gastric cancer. Consequently, their low glucose metabolism level does not necessarily correspond to a reduced risk. Investigating the metabolic characteristics of tumors in these patients is essential. Subgroup analysis reflected that the factors influencing prognosis are complex, and larger, multi-center studies would enable a more robust analysis of these subgroups and help clarify the mechanisms driving the observed differences in prognosis and SUVmax predictive value.

A higher SUVmax is also associated with the presence of microsatellite instability (MSI) status in gastric cancer [33], and the SUVmax of PET/CT imaging in microsatellite stable (MSS) gastric cancer is lower than that in MSI gastric cancer. The present study indicated that MSI-H gastric cancer was almost exclusively found in the high SUVmax group, indirectly indicating that PET/CT score can reflect the tumor mutational burden (TMB) of gastric cancer [34].

The absence of a significant difference in adjuvant chemotherapy administration between the two groups may be attributed to the impact of other clinical variables, including comorbidities, treatment adherence, and individual patient preferences. For instance, certain patients might have been deemed ineligible for adjuvant chemotherapy due to compromised health status. Usually, adjuvant chemotherapy was advocated for patients with pathological stage II or III advanced gastric cancer after undergoing D2 gastrectomy. Further indications encompass positive lymph node metastasis, poorly differentiated tumors, and positive resection margins, which may explain the comparable rates of adjuvant chemotherapy administration observed, despite variations in SUVmax and pTNM staging. Patients who underwent postoperative adjuvant chemotherapy had a poorer prognosis, which could be related to drug resistance caused by an abnormal metabolic status. Though Park et al.’s research indicated that SUVmax can serve to predict the efficacy of chemotherapy [35], our study does not support such opinion.

High SUVmax was found as an independent risk factor for operable patients with AGC, suggesting that metabolic signature is a better predictor of biologic tumor aggressiveness than its histologic signature. The current study also verified the conclusion of a previous study that high SUVmax of the primary tumor could be indicative of a high risk of recurrence in patients with AGC [36]. As a non-invasive examination, ^18^F-FDG PET/CT can provide biological information preoperatively, indirectly reflecting tumor size and TNM stage through tumor metabolism. Thus, using PET/CT as a method to predict prognosis before surgery is highly suggested. Traditionally, the depth of tumor invasion and the extent of lymph node metastasis were considered as the primary prognostic factors for patients with gastric cancer and as key components of TNM staging [37]. The present study demonstrated that integrating the SUVmax value with postoperative TNM stage could promote determining the prognosis of AGC following radical surgery.

This retrospective study has certain limitations. While multivariate analysis was conducted to mitigate potential biases and confounding variables, it is noteworthy that selection bias and other confounding factors could still influence the findings of the study.

## 5. Conclusions

In conclusion, high preoperative SUVmax could indicate more invasive clinicopathological features as well as a high expression level of GLUT-1, and it could be associated with poor PFS and OS in patients with resectable AGC.

## Figures and Tables

**Table 1:** Comparisons of clinicopathological characteristics according to the level of SUVmax.

Characteristic	Total (n = 182)	High SUVmax (n = 90)	Low SUVmax (n = 92)	χ^2^-Value	*p*-Value
Age, years, mean ± SD	62.43 ± 12.218	68.68 ± 8.694	56.32 ± 12.112		**0.006**
Age, n (%)					**<0.001**
<65	96 (52.7)	25 (27.8)	71 (77.2)	44.537	
≥65	86 (47.3)	65 (72.2)	21 (22.8)		
Sex, n (%)				4.558	**0.033**
Male	107 (58.8)	60 (66.7)	47 (51.1)		
Female	75 (41.2)	30 (33.3)	45 (48.9)		
GLUT-1 expression ^a^, n (%)				86.046	**<0.001**
Negative	89 (53.9)	14 (17.3)	75 (89.3)		
Positive	76 (46.1)	67 (82.7)	9 (10.7)		
TNM stage (AJCC 8th edition), n (%)				17.541	**0.004**
IB	18 (9.9)	2 (2.2)	16 (17.4)		
IIA	35 (19.2)	13 (14.4)	22 (23.9)		
IIB	31 (17.0)	17 (18.9)	14 (15.2)		
IIIA	47 (25.8)	28 (31.1)	19 (20.7)		
IIIB	38 (20.9)	21 (23.3)	17 (18.5)		
IIIC	13 (7.1)	9 (10.0)	4 (4.3)		
pT stage, n (%)				11.393	**0.003**
T2	41 (22.5)	11 (12.2)	30 (32.6)		
T3	71 (39.0)	42 (46.7)	29 (31.5)		
T4	70 (38.5)	37 (41.1)	33 (35.9)		
pN stage, n (%)				13.241	**0.010**
N0	59 (32.4)	18 (20.0)	41 (44.6)		
N1	28 (15.4)	16 (17.8)	12 (13.0)		
N2	41 (22.5)	26 (28.9)	15 (16.3)		
N3a	37 (20.3)	20 (22.2)	17 (18.5)		
N3b	17 (9.3)	10 (11.1)	7 (7.6)		
Lauren classification ^b^, n (%)				45.742	**<0.001**
Intestinal	40 (23.2)	34 (40.5)	6 (6.8)		
Diffuse	45 (26.2)	6 (7.1)	39 (44.3)		
Mixed	87 (50.6)	44 (52.4)	43 (48.9)		
Signet-ring cell carcinoma component, n (%)				28.860	**<0.001**
No	136 (74.7)	83 (92.2)	53 (57.6)		
Yes	46 (25.3)	7 (7.8)	39 (42.4)		
Differentiation ^c^, n (%)				3.008	0.083
Moderately differentiated	24 (14.5)	16 (19.3)	8 (9.8)		
Poorly differentiated	141 (85.5)	67 (80.7)	74 (90.2)		
MSI-H, n (%)				25.122	**<0.001**
No	157 (86.3)	66 (73.3)	91 (98.9)		
Yes	25 (13.7)	24 (26.7)	1 (1.1)		
HER2, n (%)				5.145	**0.023**
Negative	168 (92.3)	79 (87.8)	89 (96.7)		
Positive	14 (7.7)	11 (12.2)	3 (3.3)		
Type of resection, n (%)				2.676	0.102
Subtotal or distal resection	110 (60.4)	49 (54.4)	61 (66.3)		
Total gastrectomy	72 (39.6)	41 (45.6)	31 (33.7)		
Adjuvant chemotherapy, n (%)				0.547	0.459
No	90 (49.5)	47 (52.2)	43 (46.7)		
Yes	92 (50.5)	43 (47.8)	49 (53.3)		
Tumor size, n (%)				50.018	**<0.001**
<5 cm	117 (64.3)	35 (38.9)	82 (89.1)		
≥5 cm	65 (35.7)	55 (61.1)	10 (10.9)		

^a^ 17 cases are missing. ^b^ 10 cases are missing. ^c^ 17 cases are missing. *p*-Values of <0.05 have been bolded.

**Table 2:** Subgroup analysis of tumor progression rates between high and low SUVmax groups.

Variables	n (%)	High SUVmax	Low SUVmax	HR (95%CI)	*p*-Value	*p* for Interaction
All patients	182 (100.00)	38/90	23/92	0.51 (0.30~0.86)	**0.011**	
Age						0.717
<65	96 (52.75)	10/25	16/71	0.52 (0.24~1.14)	0.104	
≥65	86 (47.25)	28/65	7/21	0.62 (0.27~1.44)	0.267	
Sex						0.259
Female	75 (41.21)	11/30	13/45	0.73 (0.33~1.64)	0.448	
Male	107 (58.79)	27/60	10/47	0.39 (0.19~0.80)	**0.011**	
Stage						**0.005**
IB/IIA	53 (29.12)	7/15	3/38	0.10 (0.02~0.39)	**0.001**	
IIB/III	129 (70.88)	31/75	20/54	0.89 (0.51~1.56)	0.682	
T stage						0.674
T2	41 (22.53)	4/11	6/30	0.43 (0.12~1.54)	0.195	
T4/T3	141 (77.47)	34/79	17/62	0.58 (0.32~1.03)	0.063	
Lymph node metastasis						0.066
No	59 (32.42)	8/18	6/41	0.24 (0.08~0.69)	**0.008**	
Yes	123 (67.58)	30/72	17/51	0.76 (0.42~1.38)	0.363	
Lauren classification						0.909
Diffuse	45 (26.16)	3/6	11/39	0.53 (0.15~1.90)	0.326	
Intestinal	40 (23.26)	11/34	1/6	0.44 (0.06~3.42)	0.433	
Mixed	87 (50.58)	23/44	10/43	0.33 (0.16~0.70)	**0.004**	
SRCC						0.475
No	136 (74.73)	33/83	12/53	0.47 (0.24~0.91)	**0.026**	
Yes	46 (25.27)	5/7	11/39	0.32 (0.11~0.93)	**0.037**	
Differentiation						0.639
Low	141 (85.45)	30/67	21/74	0.55 (0.31~0.96)	**0.034**	
Mid	24 (14.55)	5/16	1/8	0.33 (0.04~2.86)	0.316	
MSI-H						0.996
No	157 (86.26)	28/66	23/91	0.53 (0.30~0.92)	**0.023**	
Yes	25 (13.74)	10/24	0/1	0.00 (0.00~Inf)	0.999	
HER2						**0.004**
Negative	168 (92.31)	34/79	20/89	0.44 (0.25~0.76)	**0.003**	
Positive	14 (7.69)	4/11	3/3	4.52 (0.98~20.80)	0.053	
Type of resection						0.362
Subtotal or distal resection	110 (60.44)	18/49	12/61	0.44 (0.21~0.92)	**0.030**	
Total gastrectomy	72 (39.56)	20/41	11/31	0.70 (0.34~1.46)	0.344	
Adjuvant chemotherapy						0.385
No	90 (49.45)	18/47	7/43	0.36 (0.15~0.86)	**0.022**	
Yes	92 (50.55)	20/43	16/49	0.60 (0.31~1.17)	0.133	
Tumor size						**0.043**
<5 cm	117 (64.29)	16/35	18/82	0.39 (0.20~0.78)	**0.007**	
≥5 cm	65 (35.71)	22/55	5/10	1.34 (0.51~3.55)	0.553	

HR = hazard ratio, CI = confidence interval. The hazard ratios and associated *p*-values were calculated using Cox proportional hazards models. *p* for interaction refers to the statistical significance of the interaction term between the SUVmax group (high vs. low) and the subgroup variable. *p*-Values of <0.05 have been bolded.

**Table 3:** Univariate and multivariate COX regression assessing associations between parameters and PFS. (*p*-Values of <0.05 have been bolded).

Variable	Univariate Analysis		Multivariate Analysis	
	HR (95%CI)	*p*-Value	HR (95%CI)	*p*-Value
Age		0.051		
<65	1			
≥65	1.66 (0.998~2.760)			
SUVmax		**0.011**		**0.042**
High	1		1	
Low	0.509 (0.303~0.856)		0.565 (0.326~0.979)	
Sex		0.634		
Male	1			
Female	0.883 (0.528~1.475)			
TNM stage (AJCC 8th edition)		**0.001**		**0.036**
I/II	1		1	
III	2.565 (1.475~4.461)		3.049 (1.076~8.639)	
pT stage		0.114		
T2	1			
T3/T4	1.731 (0.876~3.417)			
Lymph node metastasis		**0.039**		0.259
No	1		1	
Yes	1.877 (1.032~3.415)		0.522 (0.169~1.613)	
Lauren classification		0.502		
Intestinal	1			
Non-intestinal	1.243 (0.659~2.343)			
SRCC component		0.784		
No	1			
Yes	1.083 (0.612~1.918)			
Differentiation		0.308		
Moderately differentiated	1			
Poorly differentiated	1.552 (0.666~3.618)			
MSI-H		0.277		
No	1			
Yes	1.456 (0.739~2.869)			
HER2				
Negative	1	0.204		
Positive	1.666 (0.758~3.664)			
Type of resection		**0.020**		0.243
Subtotal or distal resection	1		1	
Total gastrectomy	1.833 (1.102~3.048)		1.369 (0.808~2.319)	
Adjuvant chemotherapy		**0.041**		0.060
No	1		1	
Yes	1.710 (1.023~2.857)		1.654 (0.979~2.792)	
Tumor size		0.052		
<5 cm	1			
≥5 cm	1.650 (0.995~2.737)			

## Data Availability

The datasets used and analyzed during the current study are available from the corresponding author on reasonable request.
